# Genetic Variation at the *BDNF* Locus: Evidence for Association with Long-Term Outcome after Ischemic Stroke

**DOI:** 10.1371/journal.pone.0114156

**Published:** 2014-12-03

**Authors:** Tara M. Stanne, Anna Tjärnlund-Wolf, Sandra Olsson, Katarina Jood, Christian Blomstrand, Christina Jern

**Affiliations:** 1 Institute of Biomedicine, Section for Clinical Genetics, the Sahlgrenska Academy at University of Gothenburg, Gothenburg, Sweden; 2 Institute of Neuroscience and Physiology, Section for Clinical Neuroscience and Rehabilitation, the Sahlgrenska Academy at University of Gothenburg, Gothenburg, Sweden; Charité-Universitätsmedizin Berlin, Germany

## Abstract

**Background and Purpose:**

Rates and extent of recovery after stroke vary considerably between individuals and genetic factors are thought to contribute to post-stroke outcome. Brain-derived neurotrophic factor (BDNF) plays important roles in brain plasticity and repair and has been shown to be involved in stroke severity, recovery, and outcome in animal models. Few clinical studies on BDNF genotypes in relation to ischemic stroke have been performed. The aims of the present study are therefore to investigate whether genetic variation at the BDNF locus is associated with initial stroke severity, recovery and/or short-term and long-term functional outcome after ischemic stroke.

**Methods:**

Four *BDNF* tagSNPs were analyzed in the Sahlgrenska Academy Study on Ischemic Stroke (SAHLSIS; 600 patients and 600 controls, all aged 18–70 years). Stroke severity was assessed using the NIH Stroke Scale (NIHSS). Stroke recovery was defined as the change in NIHSS over a 3-month period. Short- and long-term functional outcome post-stroke was assessed using the modified Rankin Scale at 3 months and at 2 and 7 years after stroke, respectively.

**Results:**

No SNP was associated with stroke severity or recovery at 3 months and no SNP had an impact on short-term outcome. However, rs11030119 was independently associated with poor functional outcome 7-years after stroke (OR 0.66, 95% CI 0.46–0.92; *P* =  0.006).

**Conclusions:**

BDNF gene variants were not major contributors to ischemic stroke severity, recovery, or short-term functional outcome. However, this study suggests that variants in the BDNF gene may contribute to poor long-term functional outcome after ischemic stroke.

## Introduction

Not only is stroke the second leading cause of death in developed countries, but it is also the leading cause of disability in adults [1]. There are two main causes of stroke, ischemia and hemorrhage, with ischemia being the most common (85% of all strokes in Western populations). There is a substantial inter-subject variability in outcome after stroke, beyond what is expected from known predictors such as age and stroke severity, and genetic factors are likely to play a role [2]. However, little is known about which genes are involved in poor outcome, especially in the long-term.

Brain derived neurotrophic factor (BDNF) is the most abundant neurotrophin in the brain. This growth factor plays important roles in diverse aspects of brain plasticity and repair [3] and shows neuroprotective effects in experimental models of stroke [4]–[6], as well as involvement in functional recovery and post-injury regeneration [7]. The most well studied genetic variant at the BDNF locus comprises the common Val^66^Met polymorphism (rs6265), which affects intracellular BDNF trafficking and activity-dependent secretion of BDNF in neurons [8], [9]. In human subjects, this polymorphism has been associated with cortical and hippocampal-dependent plasticity, some forms of learning and memory performance, as well as changes in brain morphology [9]–[12].

While this and other *BDNF* variants have received much attention in recent years in relation to a variety of diseases and outcome measures such as neuropsychiatric disorders, Alzheimer's [13], multiple sclerosis [14], and Parkinson's [15], clinical studies in stroke are limited. At current, it is unclear whether polymorphisms in BDNF play a role in stroke severity, recovery and functional outcome after stroke.

It has been suggested that another protein involved in neuronal repair, regeneration and survival, apolipoprotein E (ApoE), may differentially affect outcome in hemorrhagic and ischemic stroke [16]. Few studies on BDNF have separated these pathological types in analyses. Cramer *et al.* suggested a possible association for a variant in *BDNF* (rs6265) and recovery 1 month after stroke, however this finding was for total stroke [17]. When looking at studies on ischemic stroke (up to 3 months post-stroke), half reported no association [17], [18] and half reported an association [19], [20], whereas a study on subarachnoid hemorrhage reported an association ([21], see Table 1 for an overview). As far as we are aware, only one study has shown that *BDNF* variants are associated with long-term outcome (up to 1 years post-ischemic stroke, [19]), and no study has yet looked at functional outcome exceeding 1 year after ischemic stroke. The objective of the present study is therefore to investigate whether genetic variation at the *BDNF* locus is associated with stroke severity, recovery, and short-term (3-month) and long-term (2- and 7-year) functional outcome after ischemic stroke.

**Table 1:** Literature summary of *BDNF* gene variants and functional outcome after stroke.

Author	Year	Nr cases	Race	Outcome timepoint	mRS 0-1 vs 2-6?	Association w outcome?
Ischemic only						
Cramer [17]	2012	197	W 82%; A 14%; O 4%	1 m, 3 m	yes	No, at both time points
Mirowska- Guzel [18]	2012	498	W	1 m	*n/a	No
Kim [19]	2012	286	A	14 d, 1 y	yes	Yes, at both time points
Zhao [20]	2013	308	A	3 m	yes	Yes
Ischemic + Hemorrhagic						
Manso [29]	2012	546	W	3 m	no, 0 vs 1–6	No
Cramer [17]	2012	255	W 82%; A 14%; O 4%	1m, 3m	yes	No, at both time points
Mirowska- Guzel [18]	2012	554	W	1 m	*n/a	No
Subarachnoid hemorrhage						
Siironen [21]	2007	105	W	3m	**n/a	Yes

W, Western descent; A, Asian; O, Other; * The Oxford Handicap Scale "Rankin" (RS) was used to measure outcome; ** The Glasgow Outcome Scale was used.

## Materials and Methods

### Study population

The sample comprised participants from the Sahlgrenska Academy Study on Ischemic Stroke (SAHLSIS), which includes 600 patients of Western decent with ischemic stroke and 600 controls aged 18-70 years and has been described in detail elsewhere [22]. The upper age limit of 70 years was chosen based on studies showing that the influences of genetic factors are more pronounced in younger individuals [23]. Etiologic subtypes of ischemic stroke were classified according to the TOAST criteria [24] with minor modifications as previously described [25].

### Ethics statement

Informed consent was obtained from all participants prior to enrollment. For participants who were unable to communicate, consent was obtained from their next-of-kin. When written consent could not be obtained, verbal consent was carefully documented for each participant or next-of-kin. Reasons for obtaining verbal consent only included that the participant died before written consent could be obtained or was not able to fill in the written consent form and no relative was present who could assist. This study, including the procedure for obtaining consent, was approved by the IRB, i.e. the local ethics committee of the University of Gothenburg (Dnr: Ö469–99).

### Definition of vascular risk factors

Hypertension was defined by pharmacological treatment for hypertension, systolic blood pressure (SBP) ≧ 160 mm Hg, and/or diastolic blood pressure (DBP) ≧ 90mm Hg. Diabetes mellitus was defined by diet or pharmacological treatment, fasting plasma glucose ≧ 7.0 mmol/L, or fasting blood glucose ≧ 6.1 mmol/L. Hyperlipidemia was defined as pharmacological treatment, total fasting serum cholesterol level > 5.0 mmol/L, and/or LDL > 3.0 mmol/L. Smoking habit was coded as current versus never or former (smoking cessation at least one year before inclusion in the study). Information regarding vascular risk factors was registered in controls and at inclusion as well as at the 3-month follow-up in patients.

### Outcome measures

Maximum stroke severity within the first 7 days after the stroke was scored using the Scandinavian Stroke Scale (SSS; missing scores for 3 patients). As the NIH Stroke Scale (NIHSS) is more commonly used, an algorithm was used to convert the SSS into the NIHSS (25.68–0.43*SSS, [26]). As NIHSS scores were skewed and could not be transformed to normal distribution, it was dichotomized for binary logistic regression analysis. The cut-off for mild and severe stroke groups was set between 6 and 7 to be consistent with another study on BDNF [20].

Stroke severity was scored by a stroke neurologist again after 3 months (missing scores for 54 patients). Recovery was defined as change in NIHSS from acute stroke to 3-month post-stroke (ΔNIHSS; missing scores for 55 patients). This time point was chosen as most spontaneous recovery tends to occur within the first 3 months after stroke onset [2]. As the recovery score was also skewed and could not be transformed to normal distribution, it was dichotomized for binary logistic regression analysis. The cut-off was set at 0, with a negative value indicating clinical improvement and ≧0 indicating no change or clinical deterioration.

Functional outcome was assessed 3 months, 2 years and 7 years post-stroke by the modified Rankin Scale (mRS, [27]). At the 3-month follow-up, mRS was scored by one stroke neurologist (missing scores for 31 patients). At the 2-year follow-up, all surviving patients (or when relevant their next-of kin) were interviewed by one study nurse who was trained in stroke medicine and also specifically trained to score mRS (n = 25 patients had died and are coded mRS = 6; missing scores for 7 patients). Surviving patients were also interviewed by the research nurse at 7-year follow-up (n = 98 patients had died and are coded mRS = 6; missing scores for n = 115). Given that there are many confounding factors related to death after 7-years, the primary analysis was performed on the 7-year mRS data excluding the patients that died after 2-year follow-up (n = 73 patients were excluded). A secondary analysis that included all patients was also performed, with similar findings (please see Table S2). Because the mRS is an ordinal scale without proportional odds it was dichotomized. Here, the cut-off for the good and poor functional recovery groups was set between 1 and 2, as this cut-off has been used in the majority of studies on BDNF, which facilitates comparison (see overview Table 1). This cut-off distinguishes the clinical outcome of having no/mild disability versus a disability.

### Genotyping

Genotype data from the CEU population in the HapMap project (release 23) was entered into HaploView software package 4.2 to select tag SNPs that would capture unmeasured variation over the *BDNF* gene with an r^2^>0.80. Using the “solid spine of LD” setting, 4 SNPs were selected by the “Tagger” algorithm to capture SNPs with MAF>0.10. The four chosen SNPs capture 27 of 31 (87%) alleles at r^2^>0.80, with a mean max r^2^ of 0.94. A schematic presentation of the 4 SNPs is shown in Figure S1. Genotyping was performed with the GoldenGate Assay (Illumina Inc., San Diego, CA, USA). All genotyping was performed blinded to NIHSS and mRS scores. The genotyping success rates were between 97–100%.

### Statistics

The NIHSS scores were skewed and could not be transformed to normal distributions. Therefore the non-parametric Kruskal-Wallis test was used to compare NIHSS levels between ischemic stroke subtypes.

All SNPs were in Hardy-Weinberg equilibrium in both patients and controls. Additive models in binary logistic regression were performed to determine the associations between *BDNF* SNPs and stroke severity (NIHSS score ≤6 mild versus>6 severe), stroke recovery (ΔNIHSS <0 improvement versus ≧0 no change or deterioration), and functional outcome (mRS 0–1 good versus 2–6 poor). The majority of studies on rs6265 have used a dominant genetic model since the minor allele is rare in populations of Western descent (only 30% of persons carry at least one minor allele and approx. 4% are homozygous, [28]). Thus, dominant models were also calculated for this SNP, which yielded comparable results (please see Table S1). For stroke severity and stroke recovery the regression analyses were adjusted for age and sex (model A), as well as hypertension, diabetes mellitus, hyperlipidemia, smoking and etiologic stroke subtype (model B). For functional outcome the two regression models included the same covariates as above, with the exception of model B which also included initial stroke severity.

Assuming a multiplicative genetic model and a frequency of 0.30 for the high risk allele, the odds ratio (OR) that can be detected with 80% power at the 5% level is 1.25 for stroke severity, 1.27 for stroke recovery and 1.38, 1.36 and 1.48 for functional outcome after 3 months, 2 years and 7 years, respectively.

All statistical analyses were performed using SPSS for Windows, v20 (IBM SPSS Inc, NY, USA). The statistical significance level was 0.05 and *P*-values were two-tailed. Because all SNPs were located within one LD block (see Figure S1), no correction for multiple testing was performed. The manuscript was prepared according to the STROBE guidelines.

## Results

Baseline characteristics for SAHLSIS samples have been described previously and are summarized in Table 2 [22]. As expected, the traditional vascular risk factors hypertension, diabetes, hyperlipidemia and smoking were all over-represented in overall ischemic stroke and the four main etiologic subtypes as compared to controls. Initial stroke severity, as measured by NIHSS, was significantly different across TOAST subtypes in the acute phase (χ^2^ = 11.4, *P* = 0.010). Patients with the subtype small vessel disease (SVD) had the mildest strokes and patients with cardioembolic (CE) stroke had the most severe strokes. Stroke severity after 3 months was not subtype-dependent (χ^2^ = 7.3, *P* = 0.062).

**Table 2:** Baseline characteristics of the Sahlgrenska Academy Study on Ischemic Stroke (SAHLSIS).

	Control	Ischemic stroke	LVD	SVD	CE	Cryptogenic stroke
	(n = 600)	(n = 600)	(n = 73)	(n = 124)	(n = 98)	(n = 162)
Median age, yr (IQR)	58 (51–64)	58 (51–64)	60 (57–64)	59 (54–64)	61 (53–65)	56 (48–61)
Male, n (%)	385 (64)	385 (64)	54 (74)	77 (62)	66 (67)	95 (59)
Hypertension, n (%)	224 (37)	354 (59)	44 (63)	89 (72)	50 (52)	87 (54)
Diabetes, n (%)	33 (5)	114 (19)	25 (34)	26 (21)	19 (19)	23 (14)
Hyperlipidemia, n (%)	403 (67)	413 (69)	53 (81)	77 (71)	73 (82)	107 (71)
Current smoking, n (%)	109 (18)	233 (39)	39 (54)	54 (43)	34 (35)	60 (37)
NIHSS ac, m (IQR)	N/A	2.9 (1.6–7.2)	3.3 (1.2–11.5)	2.2 (1.3–4.1)	3.8 (1.2–11.1)	2.5 (1.2–6.4)
NIHSS 3m, m (IQR)	N/A	0.7 (0.7–2.5)	1.6 (0.7–3.3)	0.7 (0.7–2.0)	1.2 (0.7–3.3)	0.7 (0.7–2.5)
ΔNIHSS, m (IQR)	N/A	−1.3 (−3.9 to 0)	−1.3 (−3.0 to 0)	−0.8 (−2.6 to 0)	−1.7 (−4.7 to 0)	−1.3 (−4.1 to 0)
rs6265, MAF	0.19	0.18	0.17	0.19	0.24	0.14
rs11030107, MAF	0.27	0.24	0.24	0.25	0.25	0.26
rs11030119, MAF	0.31	0.29	0.31	0.30	0.27	0.30
rs2049046, MAF	0.44	0.47	0.47	0.45	0.42	0.51

Data are shown as median (m) and interquartile range (IQR), number (n) and percentage, or minor allele frequency (MAF). LVD, large vessel disease; SVD, small vessel disease; CE, cardioembolic stroke; NIHSS ac, initial stroke severity during the acute phase; NIHSS 3m, stroke severity after 3 months; ΔNIHSS, recovery defined as change in NIHSS from acute to 3-month post-stroke, with a negative value indicating clinical improvement.

### *BDNF* genotypes and stroke severity

Of the 597 patients with acute NIHSS scores, 444 individuals were classified as having a mild stroke and 153 as having a severe stroke. Table 3 shows the distribution of the *BDNF* genotypes according to stroke severity. Logistic regression analyses were then used to determine the effect of genetic variables on stroke severity after adjusting for age and sex (model A), as well as traditional risk factors and stroke subtype (model B). The minor alleles of rs6265 and rs2049046 had a trend toward more severe stroke, whereas rs11030107 and rs11030119 had a trend towards milder stroke. However, no significant association was found between severity of stroke and any of the *BDNF* variants.

**Table 3:** *BDNF* genotype frequency distribution and association with stroke severity during the acute phase, as measured by the NIHSS.

SNP	Genotype	Mild (< = 6) n = 444	Severe (>6) n = 153
rs6265	GG, n (%)	291 (68)	89 (60)
	GA, n (%)	127 (30)	54 (37)
	AA, n (%)	11 (3)	4 (3)
	OR (95% CI)	ref	1.29 (0.91–1.82)^a^
		ref	1.10 (0.74–1.62)^b^
rs11030107	AA, n (%)	254 (58)	95 (62)
	AG, n (%)	155 (35)	53 (35)
	GG, n (%)	32 (7)	5 (3)
	OR (95% CI)	ref	0.79 (0.58–1.08)^a^
		ref	0.88 (0.63–1.22)^b^
rs11030119	GG, n (%)	221 (50)	84 (55)
	GA, n (%)	176 (40)	60 (39)
	AA, n (%)	44 (10)	9 (6)
	OR (95% CI)	ref	0.81 (0.60–1.08)^a^
		ref	0.85 (0.62–1.17)^b^
rs2049046	TT, n (%)	120 (27)	34 (22)
	TA, n (%)	226 (52)	90 (59)
	AA, n (%)	95 (21)	29 (19)
	OR (95% CI)	ref	1.06 (0.81–1.39)^a^
		ref	1.12 (0.83–1.50)^b^

OR, odds ratio; 95% CI, confidence interval; ^a^Adjusted for age and sex; ^b^Adjusted for age, sex, hypertension, diabetes, hyperlipidemia, smoking and TOAST subtype.

### *BDNF* genotypes and recovery after stroke (ΔNIHSS)

Recovery was expressed as change in NIHSS score from acute stroke to 3 months post-stroke, with a negative value indicating clinical improvement. Of the 545 patients with ΔNIHSS scores, 380 individuals were classified as having improved and 165 as having deteriorated or remained stable. Table 4 shows the distribution of *BDNF* genotypes according to stroke recovery. Interestingly, the minor allele of rs6265 and rs2049046 had a trend towards improvement between baseline and 3 months while the minor allele of rs11030107 and rs11030119 had a trend towards deterioration over time. These results are compatible with another study, which showed that carriers of the rs6265 minor allele scored higher in NIHSS at baseline indicating more severe stroke, but improved more within 30 days than non-carriers [18]. However, despite these trends, no polymorphism was significantly associated with recovery after 3 months in either regression model (Table 4).

**Table 4:** *BDNF* genotype frequency distribution and association to recovery as measured by the change in NIHSS score from acute stroke to 3 months post-stroke (ΔNIHSS).

SNP	Genotype	Improvement (<0) n = 380	No change/deterioration (> = 0) n = 165
rs6265	GG, n (%)	238 (65)	117 (74)
	GA, n (%)	121 (33)	37 (23)
	AA, n (%)	8 (2)	5 (3)
	OR (95% CI)	ref	0.75 (0.51–1.09)^a^
		ref	0.79 (0.54–1.17)^b^
rs11030107	AA, n (%)	228 (60)	91 (55)
	AG, n (%)	127 (34)	61 (37)
	GG, n (%)	23 (6)	12 (7)
	OR (95% CI)	ref	1.17 (0.87–1.57)^a^
		ref	1.12 (0.83–1.52)^b^
rs11030119	GG, n (%)	201 (53)	76 (46)
	GA, n (%)	143 (38)	72 (44)
	AA, n (%)	34 (9)	16 (10)
	OR (95% CI)	ref	1.19 (0.90–1.57)^a^
		ref	1.19 (0.89–1.58)^b^
rs2049046	TT, n (%)	96 (25)	41 (25)
	TA, n (%)	198 (52)	90 (55)
	AA, n (%)	84 (22)	33 (20)
	OR (95% CI)	ref	0.96 (0.73–1.25)^a^
		ref	0.94 (0.71–1.24)^b^

OR, odds ratio; 95% CI, confidence interval; ^a^Adjusted for age and sex; ^b^Adjusted for age, sex, hypertension, diabetes, hyperlipidemia, smoking and TOAST subtype.

### *BDNF* genotypes and short-term functional outcome (3 months)

**Figure 1:**
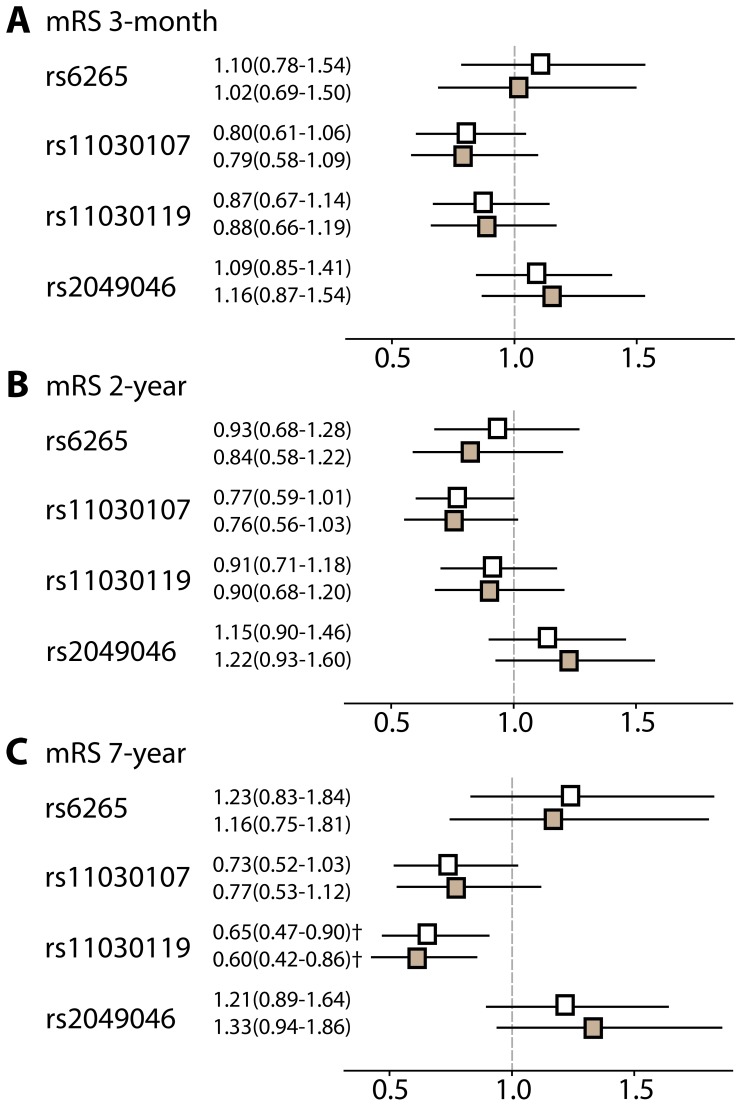
Odds ratios and 95% confidence intervals for the associations between SNPs in *BDNF* and poor functional outcome after stroke as measured by mRS ≧2 at 3 months (A), 2 years (B), and 7 years (C) post-stroke. White boxes, adjusted for age and sex; grey boxes adjusted for age, sex, smoking, diabetes, hypertension, hyperlipidemia, TOAST subtype and NIHSS during the acute phase. † *P*<0.01 compared with good outcome after stroke. Patients that died after the 2-year follow-up were excluded from the 7-year analyses (n = 73).

*BDNF* variants were next compared in good (mRS 0–1) versus bad (mRS 2–6) functional outcome groups after 3 months post-stroke. Genotype frequencies according to outcome are presented in Table 5. Multivariate logistic regression analyses were performed to correct for age and sex (model A), as well as traditional vascular risk factors, TOAST subtype, and initial stroke severity (model B; Figure 1A). The minor alleles of rs6265 and rs2049046 had a trend towards poor outcome whereas rs11030107 and rs11030119 had a trend towards favourable outcome. The direction of the finding for rs6265 is in agreement with previous studies [17], [19]–[21], [29]. However, no significant association was observed for any SNP after short-term (3-month) follow-up (Figure 1A). Similar results were obtained for rs6265 when using a dominant model (Table S1).

**Table 5:** *BDNF* genotype percentages by stroke outcome status as measured by the modified Rankin Scale (mRS) at 3 months, 2 years and 7 years post stroke.

SNP	3-month		2-year		7-year *	
	Good	Poor	Good	Poor	Good	Poor
	n = 206	n = 362	n = 233	n = 360	n = 142	n = 270
rs6265						
GG, n (%)	134 (68)	232 (66)	145 (64)	233 (67)	93 (67)	165 (64)
GA, n (%)	60 (31)	109 (31)	76 (34)	103 (30)	43 (31)	88 (34)
AA, n (%)	3 (2)	11 (3)	4 (2)	10 (3)	2 (1)	7 (3)
rs11030107						
AA, n (%)	113 (56)	221 (61)	123 (54)	220 (61)	71 (50)	164 (61)
AG, n (%)	73 (36)	122 (34)	90 (39)	120 (33)	62 (44)	91 (34)
GG, n (%)	17 (8)	19 (5)	17 (7)	19 (5)	8 (6)	14 (5)
rs11030119						
GG, n (%)	101 (50)	192 (53)	114 (50)	185 (52)	60 (43)	147 (55)
GA, n (%)	80 (39)	143 (39)	93 (40)	145 (40)	66 (47)	105 (39)
AA, n (%)	22 (11)	27 (8)	23 (10)	29 (8)	15 (11)	17 (6)
rs2049046						
TT, n (%)	56 (28)	87 (24)	69 (30)	83 (23)	42 (30)	62 (23)
TA, n (%)	104 (51)	196 (54)	112 (49)	203 (57)	72 (51)	151 (56)
AA, n (%)	43 (21)	79 (22)	49 (20)	73 (20)	27 (19)	56 (21)

Good outcome, mRS 0–1; Poor outcome, mRS 2–6; * Patients that died after 2 years were excluded (n = 73).

### *BDNF* genotypes and long-term functional outcome (2 and 7 years)

Long-term outcome was next analysed by comparing good versus bad mRS scores after 2 and 7 years post-stroke. Genotype frequencies according to outcome at the various time points are presented in Table 5. At 2-year follow up, the trend for each SNP remained in the same direction as at the 3-month follow up, with the exception of rs6265 which had a very modest trend towards favorable outcome (Figure 1B; rs11030107 *P* = 0.057 model A and *P* = 0.076 model B). At 7-year follow up the trend for rs11030107 remained (Figure 1C) and the minor allele of rs11030119 was associated with favorable outcome, independent of other known predictors of poor outcome (*P* = 0.010 model A and *P* = 0.005 model B). In the secondary analysis, when patients that died after 2-years were included, similar results were obtained (model B, odds ratio (OR), 95% confidence interval (CI): 0.65, 0.47–0.91, *P* = 0.013; Table S2).

## Discussion

In the present study, genetic variation at the *BDNF* locus was not a major contributor to ischemic stroke severity, recovery or short-term functional outcome. However a role for BDNF in long-term functional outcome post-stroke was suggested.

### Stroke severity

Brain plasticity allows the human brain to adapt to environmental pressure and challenges including brain damage. Genetic polymorphisms are one factor that may influence the response of the brain to injury and disease. BDNF is the most abundant neurotrophin in the brain and is involved in many facets of brain function, including neuronal survival and plasticity. Several animal studies have shown that BDNF levels are increased in the brain after stroke [30], [31] and one study showed that plasma BDNF levels were positively correlated with stroke severity in rats [32]. The most well studied polymorphism in *BDNF*, rs6265 (Val^66^Met), is associated with impaired activity-dependent secretion of BDNF in neurons [9] and has been analyzed with respect to stroke severity in several clinical studies with discrepant results. Of these, one reported that patients homozygous for the major allele of rs6265 (GG) scored better on NIHSS at the time of admission for hemorrhagic stroke [18], while three studies on ischemic stroke failed to demonstrate an association between *BDNF* genotype and initial stroke severity [18]–[20]. Our observations are in agreement with these later studies on ischemic stroke. This discrepancy is likely due to the different type of strokes analyzed.

### Short-term recovery

BDNF promotes both neuro- and angiogenesis [33], [34] and has been shown to promote post-stroke recovery in animal models [35]. The rs6265 polymorphism has been demonstrated to affect learning and plasticity [9]. The study by Cramer et al included a subset of 255 stroke cases from the GAIN clinical trials. Although an association for rs6265 and 1-month recovery was detected, the association was not significant when the analysis was restricted to ischemic stroke (n = 197, [17]). Furthermore, no significant association was found for overall or ischemic stroke and 3-month recovery [17]. In line with this, when analyzing recovery based on ΔNIHSS score from baseline to 3-month follow-up in SAHLSIS, no significant association with any *BDNF* polymorphism was found. Thus, in spite of substantial experimental evidence linking BDNF to neuronal survival, plasticity, and repair, genetic association studies so far do not lend firm support for *BDNF* variation as major contributor to either ischemic stroke severity or short-term recovery after ischemic stroke.

### Short-term functional outcome

Recently, several clinical studies have analyzed rs6265 and other *BDNF* polymorphisms in relation to functional outcome after stroke (Table 1). While two Asian studies reported an association between rs6265 and 2-week and 3-month functional outcome as measured by mRS ([19] and [20], respectively), no association was observed in Western populations between BDNF variants and 1-month [18] or 3-month [17], [29] post-stroke outcome. In agreement with these latter studies, no association was detected for the mRS score 3 months after ischemic stroke in SAHLSIS. Ethnic differences in the risk allele frequencies may in part explain the positive findings in the Asian cohorts [19], [20] as the minor allele of rs6265 is much more prevalent in Asian compared to Western populations (49% vs 18–21%, [17], [19]–[21]). It is of note that the direction of the finding for the rs6265 is the same in all studies. Therefore, the lack of association in populations of Western descent may reflect decreased statistical power. However, in a small study on subarachnoid hemorrhage survivors, the minor allele of rs6265 was reported to associate with poor prognosis after 3 months as monitored by the Glasgow Outcome Scale [21]. As with ApoE, this suggests BNDF may differentially affect outcome in hemorrhagic and ischemic stroke [16].

### Long-term outcome

Adaptive neuroplasticity and remodeling occur for an extended period after stroke [36], [37] and patients often exhibit continued functional recovery for many years following their initial injury [2]. Despite this, few studies have reported on long-term outcomes after ischemic stroke. As far as we are aware, only one study has evaluated both short-term (after 2-weeks) and long-term (after 1 year) consequences after stroke for *BDNF* genotypes [19]. In that Asian study, the minor allele of rs6265 was significantly associated with poor outcome at both time points, with a stronger association observed 1-year post-stroke (1-year, OR 3.14, 95% CI 1.29–7.68 versus 2–week, OR 2.75, 95% CI 1.20–6.31, [19]). In line with this, all SNPs in our study had stronger associations at the 7-year time-point compared to the 3-month time-point, although only rs11030119 achieved statistical significance. These results suggest that *BDNF* gene variants may contribute to long-term adaptive neuroplasticity and functional repair following brain injury. Although the exact mechanism is unclear at this time, more studies are warranted.

### Strengths and limitations

Strengths of the present study include the well-characterized clinical sample, SAHLSIS, which comprises patients from the southwest of Sweden who are relatively homogenous from a genetic point of view, all of whom have suffered from ischemic stroke. Furthermore, the study is longitudinal in nature, and includes measurement of within-subject change beginning with the acute presentation through to 3-month, 2-year and 7-year follow-up. Participants in SAHLSIS were recruited consecutively, which reduces the likelihood of selection bias. A range of covariates were also considered in the analyses, including etiologic ischemic stroke subtype classification which has been demonstrated previously to influence outcome after stroke. There are also some limitations which should be considered. Biases in selection of subjects may occur in case-control design since only survivors of ischemic stroke are examined. However the stroke admission rate in Sweden is very high and the early case fatality for the age group under study is low. Although relatively large compared to other studies on *BDNF* genotypes and post-stroke outcome, the sample size is small by genetic study standards and thus the statistical power is low, which is especially true for the 7-year follow-up. Finally, replication analysis for the 7-year mRS data was not possible, as no other study on ischemic stroke has followed stroke patients this long.

## Conclusion

This is the first evaluation of the association between BDNF genotypes and long-term outcomes after ischemic stroke in a population of Western decent. Our study suggests that genetic variation in the *BDNF* region may be related to differences in long-term outcome after ischemic stroke, independent of baseline deficits, etiologic subtype, and traditional cardiovascular risk factors. However, further investigations based on larger, well-characterized samples of patients with ischemic stroke and long-term outcome are clearly warranted.

## Supporting Information

Figure S1 Schematic representation of the *BDNF* gene: the location of the exons and a graphical representation of the linkage disequilibrium (LD) structure.
(DOCX)

Table S1 Odds ratios and 95% confidence intervals for the associations between the *BDNF* SNP rs6265 and poor functional outcome after stroke as measured by mRS ≧2 at 3 months, 2 years, and 7 years post-stroke, calculated using a dominant genetic model.
(DOCX)

Table S2 Secondary analysis of 7-year follow-up, including patients that died after 2-years (n = 73). Genotype frequency distribution and associations between SNPs in *BDNF* and poor functional outcome 7-years after stroke as measured by mRS ≧2.
(DOCX)
