# Supplementary material for: Genetic Variation at the BDNF Locus: Evidence for Association with Long-Term Outcome after Ischemic Stroke
Source: PLoS One. 2014 Dec 3;9(12):e114156. doi: 10.1371/journal.pone.0114156 (PMC4254920; doi:10.1371/journal.pone.0114156)

**Figure S1.** Schematic representation of the *BDNF* gene: the location of the exons and a graphical representation of the linkage disequilibrium (LD) structure. Positions of the four selected tagSNPs are indicated in numerical order from 3’ to 5. A) LD plot for all SNPs with a minor allele frequency >0.10 in the 100kb locus. B) LD plot for the analyzed tagSNPs. The LD blocks were defined by using Haploview 4.2 and the method “Solid Spine of LD”.


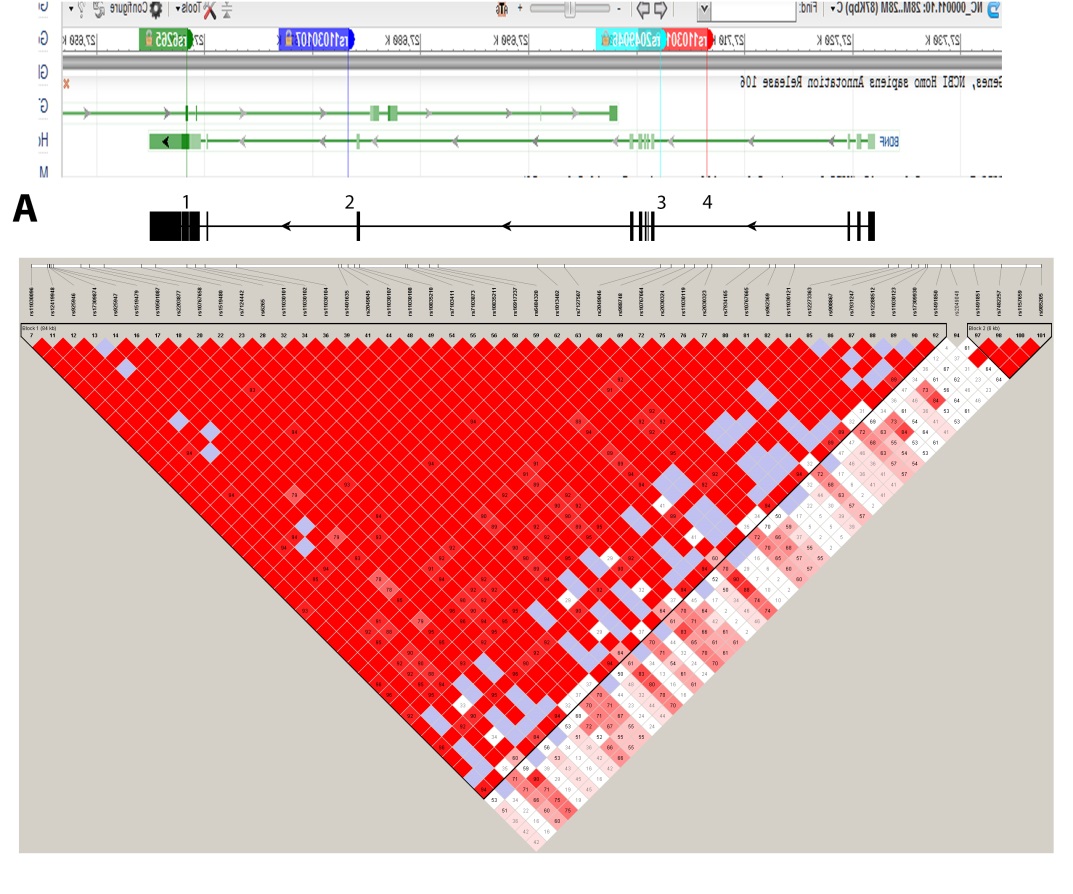


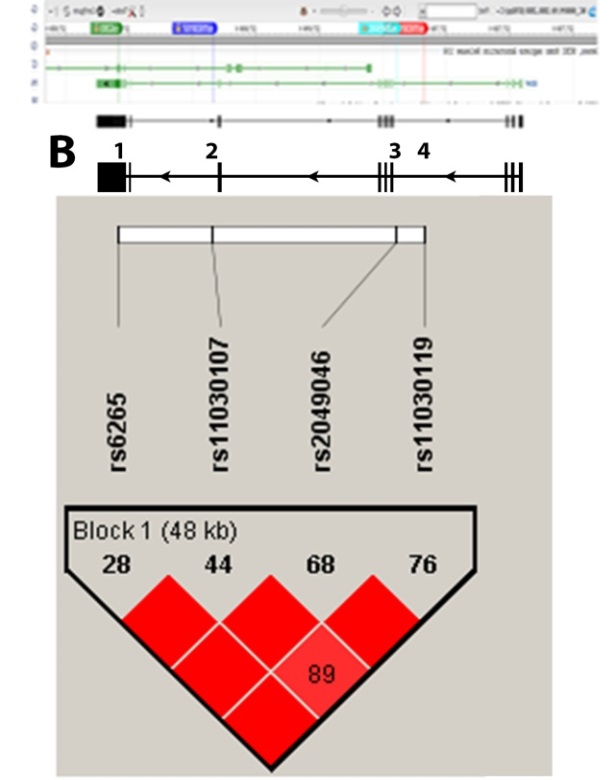

Supplement: Figure S1 — Schematic representation of the BDNF gene: the location of the exons and a graphical representation of the linkage disequilibrium (LD) structure. (DOCX) [file pone.0114156.s001.docx]
