# Supplementary material for: Genetic Variation at the BDNF Locus: Evidence for Association with Long-Term Outcome after Ischemic Stroke
Source: PLoS One. 2014 Dec 3;9(12):e114156. doi: 10.1371/journal.pone.0114156 (PMC4254920; doi:10.1371/journal.pone.0114156)
Supplement: Table S1 — Odds ratios and 95% confidence intervals for the associations between the BDNF SNP rs6265 and poor functional outcome after stroke as measured by mRS ≧2 at 3 months, 2 years, and 7 years post-stroke, calculated using a dominant genetic model. (DOCX) [file pone.0114156.s002.docx]

**Table S1:** Odds ratios and 95% confidence intervals for the associations between the *BDNF* SNP rs6265 and poor functional outcome after stroke as measured by mRS ≥2 at 3 months, 2 years, and 7 years post-stroke, calculated using a dominant genetic model.

| Model | 3 months | 2 years | 7 years* |
| --- | --- | --- | --- |
|  | OR (95% CI)  Good/ Poor | OR (95% CI)  Good/ Poor | OR (95%CI)  Good/ Poor |
|  | n=206/ 362 | n=233/ 360 | n=142/ 270 |
| A | 1.10 (0.78-1.54) | 0.94 (0.68-1.29) | 1.22 (0.82-1.82) |
| B | 0.99 (0.69-1.43) | 0.83 (0.58-1.17) | 1.13 (0.74-1.73) |

Good outcome, mRS 0-1; Poor outcome, mRS 2-6; OR, odds ratio; 95% CI, 95% confidence intervals. *Patients that died after 2 years were excluded (n=73).
