# Supplementary material for: Genetic Variation at the BDNF Locus: Evidence for Association with Long-Term Outcome after Ischemic Stroke
Source: PLoS One. 2014 Dec 3;9(12):e114156. doi: 10.1371/journal.pone.0114156 (PMC4254920; doi:10.1371/journal.pone.0114156)
Supplement: Table S2 — Secondary analysis of 7-year follow-up, including patients that died after 2-years (n = 73). Genotype frequency distribution and associations between SNPs in BDNF and poor functional outcome 7-years after stroke as measured by mRS ≧2. (DOCX) [file pone.0114156.s003.docx]

**Table S2:** Secondary analysis of 7-year follow-up, including patients that died after 2-years (n=73). Genotype frequency distribution and associations between SNPs in *BDNF* and poor functional outcome 7-years after stroke as measured by mRS ≥2.

|  | | Good  n=142 | Poor  n=343 |
| --- | --- | --- | --- |
|  | |  |  |
|  |  |  |  |
| rs6265 | GG, n (%) | 93 (67) | 215 (65) |
|  | GA, n (%) | 43 (31) | 108 (33) |
|  | AA, n (%) | 2 (1) | 8 (2) |
|  | OR (95% CI) | ref | 1.19 (0.80-1.75)^a^ |
|  |  | ref | 1.08 (0.70-1.66)^b^ |
|  |  |  |  |
| rs11030107 | AA, n (%) | 71 (50) | 205 (60) |
|  | AG, n (%) | 62 (44) | 116 (34) |
|  | GG, n (%) | 8 (6) | 21 (6) |
|  | OR (95% CI) | ref | 0.78 (0.57-1.08)^a^ |
|  |  | ref | 0.84 (0.59-1.19)^b^ |
|  |  |  |  |
| rs11030119 | GG, n (%) | 60 (43) | 182 (53) |
|  | GA, n (%) | 66 (47) | 135 (40) |
|  | AA, n (%) | 15 (11) | 25 (7) |
|  | OR (95% CI) | ref | 0.70 (0.51-0.94)^a^* |
|  |  | ref | 0.65 (0.47-0.92)^b^* |
|  |  |  |  |
| rs2049046 | TT, n (%) | 42 (30) | 82 (24) |
|  | TA, n (%) | 72 (51) | 188 (55) |
|  | AA, n (%) | 27 (19) | 72 (21) |
|  | OR (95% CI) | ref | 1.19 (0.89-1.59)^a^ |
|  |  | ref | 1.30 (0.94-1.80)^b^ |

OR, odds ratio; 95% CI, 95% confidence intervals; ^a^Adjusted for age and sex; ^b^Adjusted for age, sex, traditional risk factors, baseline NIHSS and TOAST subtype. *P<0.05 compared with the good outcome after stroke
